# Prevalence of anaemia, iron, and vitamin deficiencies in the health system in the Republic of Ireland: a retrospective cohort study

**DOI:** 10.3399/BJGPO.2023.0126

**Published:** 2024-04-17

**Authors:** Conor Cian Clancy, Leonard D Browne, Robert Gilligan, Ophelia Blake, Austin G Stack

**Affiliations:** 1 School of Medicine, University of Limerick, Limerick, Ireland; 2 Health Research Institute (HRI), University of Limerick, Limerick, Ireland; 3 Department of Biochemistry, University Hospital Limerick, Limerick, Ireland; 4 Department of Nephrology, University Hospital Limerick, Limerick, Ireland

**Keywords:** anaemia, iron deficiencies, prevalence, ageing, screening

## Abstract

**Background:**

Anaemia is a common but treatable condition that predicts adverse clinical outcomes. However, standards of anaemia management vary considerably.

**Aim:**

To estimate the prevalence of anaemia and extent of screening for common underlying causes in the healthcare system in the Republic of Ireland.

**Design & setting:**

We conducted a retrospective cohort study of 112 181 adult patients, aged ≥18 years, who had a full blood count performed in 2013, using data from the National Kidney Disease Surveillance System.

**Method:**

The prevalence of anaemia was determined across demographic and clinical subgroups, according to World Health Organization (WHO) definitions. The proportion screened for iron, vitamin B12, and folate deficiency was determined within a 3-month follow-up period and the corresponding prevalence for each deficiency determined.

**Results:**

The overall prevalence of anaemia was 12.0% (95% confidence interval [CI] = 11.8% to 12.2%) and was higher in women than men (13.2% versus 10.5%, *P*<0.001). Anaemia increased with advancing age (33.4% for those aged >75 years) and worsening kidney function (8.2%, 10.9%, 33.2%, and 63.8% for each estimated glomerular filtration rate [eGFR] categories >90, 60–89, 30–59 and <30 ml/min/1.73 m², respectively, *P*<0.001). After 3-months' follow-up, the proportion screened for iron deficiency was 11.2% based on transferrin saturation and 33.7% using serum ferritin. Screening for folate and B12 deficiency was 17.6% and 19.8%, respectively. Among screened patients, the prevalence of iron deficiency, B12, and folate deficiency was 37.0%, 6.3%, and 5.8%, respectively.

**Conclusion:**

The burden of anaemia in the healthcare system is substantial especially for older patients and those with advanced kidney disease. Low screening rates for iron, B12, and folate deficiency are common and warrant quality improvement initiatives.

## How this fits in

In the Republic of Ireland, the prevalence of anaemia and information on its underlying causes is largely unknown as data at the national or regional level are limited. In this large population-based study, we report a substantial burden of anaemia within the Irish health system. Strikingly, we report low frequencies of screening for treatable causes of anaemia, including deficiencies in vitamin B12, folate, and iron. This highlights a significant gap in care delivery programmes and emphasises the need for quality improvement initiatives.

## Introduction

Anaemia is a common and often treatable condition in older patients.^1^ It is associated with adverse health outcomes,^2–4^ especially in the setting of chronic disease.^5^ With an ageing population, the burden of anaemia is likely to increase further and adversely impact patient outcomes within health systems.^6,7^ The underlying causes of anaemia in older adults are diverse with nutritional deficiencies and chronic disease cited as common causes.^6^ The presence of anaemia is often diagnosed from routine laboratory testing by screening for serum haemoglobin concentration^8^ and in many cases the underlying cause of anaemia can be easily treated.^6,7,9^ The identification of a specific cause and subtype of anaemia is important as this informs treatment planning. Treatment has been shown to have a significant improvement on quality of life^9^ and is likely to yield significant cost-savings.^7,10^

Clinical guidelines have recommended that the appropriate investigation of anaemia subtype should include the following: an assessment of iron status (serum ferritin, transferrin receptor saturation [TSAT]); screening for B12 or folate deficiency, and measuring C-reactive protein (CRP).^6^ However, recent studies have suggested that the investigation of anaemia subtype is uncommon and that the standards of anaemia management vary considerably.^11,12^ Furthermore, it is unclear to what extent common nutritional deficiencies of iron, B12, and folate are screened for among patients with confirmed anaemia, the magnitude of these deficiencies, and whether variability exists across vulnerable subgroups. Understanding current clinical practices and identifying gaps in care delivery would provide a valuable starting point for quality improvement programmes.

This study aimed to determine the prevalence and severity of anaemia in the health system in the Republic of Ireland across demographic and clinical subgroups. Additionally, it aimed to evaluate the extent of screening for iron, B12, and folate deficiency and quantify the degree of deficiency within a 3-month follow-up period.

## Method

### Study design

We conducted a retrospective cohort study using data from the National Kidney Disease Surveillance System (NKDSS). The NKDSS monitors the health and outcomes of patients with kidney disease in the health system in the Republic of Ireland. The principal data sources include regional laboratory information systems, which capture a comprehensive list of laboratory results from inpatients and outpatients within a designated health region; dialysis registers that capture clinical data on patients who progress to end-stage kidney disease (ESKD); and mortality data files from the national Central Statistics Office (CSO). The final merged dataset captured information on demographic characteristics, county of residence, clinical location of blood test capture (clinical setting), multiple laboratory measures of health status, dialysis indicator variables, and death.^13–15^

We identified all adult patients, aged ≥18 years, in the Midwest health region, who had a full blood count performed as part of routine care in 2013. We excluded patients who were receiving renal replacement therapy (RRT) and those who had missing data for age, sex, healthcare setting, or estimated glomerular filtration rate (eGFR). We restricted the study to patients who had their first measurement of serum haemoglobin concentration performed at entry to the healthcare system in 2013 (Supplementary Figure S1). The characteristics of patients who were excluded are shown in Supplementary Table S8. We determined the extent of investigation of anaemia in the 3-month period following the date of first haemoglobin measurement (anaemia diagnosis) to explore the use of screening tests for anaemia subtype, including markers of iron deficiency, folate and B12 deficiency, nutritional indicators, measures of kidney function, liver function, and inflammation. All blood samples were taken at University of Limerick Hospitals Group facilities and associated primary care centres as part of routine clinical care.

### Definitions

Anaemia and its severity were defined according to World Health Organization (WHO) criteria^16^ and are presented in Table 1. Locally derived reference ranges were used to define anaemia by mean corpuscular volume (MCV). The Kidney Disease Improving Global Outcomes (KDIGO) guidelines were used to define chronic kidney disease (CKD) as eGFR <60 ml/min/1.73 m².^17^ Blood samples for haematology were analysed using the Siemens ADVIA 21210i system. All other blood samples were analysed using the Roche cobas system.

**Table 1.** Threshold values for defining anaemia, iron, B12, and folate deficiency

Condition	Type	Definition
Anaemia^a^		Men: Hb <13 g/dl
		Women: Hb <12 g/dl
		Pregnant women: Hb <11 g/dl
Anaemia severity	Mild	Men: Hb 11–12.9 g/dl
		Women: Hb 11–11.9 g/dl
		Pregnant women: Hb 10–10.9 g/dl
	Moderate	Men: Hb 8.0–10.9 g/dl
		Women: Hb 8.0–10.9 g/dl
		Pregnant women: Hb: 7.0–9.9 g/dl
	Severe	Men: Hb <8 g/dl
		Women: Hb <8 g/dl
		Pregnant women: Hb <7 g/dl
Anaemia type	Microcytic	MCV <76 fl
	Normocytic	MCV 76–96 fl
	Macrocytic	MCV >96 fl
Iron deficiency^b^	Absolute iron deficiency	SF <30 ng/ml
	Functional iron deficiency	SF 30–99 ng/ml OR
		SF 100–299 ng/ml AND TSAT <20%
B12 deficiency		B12<200 pg/ml
Folate deficiency		Folate <3 ng/ml

^a^ Anaemia thresholds from World Health Organization (WHO).^16^ ^b^ Iron deficiency definition for a general population.^21^ Hb = haemoglobin. MCV = median corpuscular volume. SF = serum ferritin. TSAT = transferrin saturation.

### Statistical methods

Baseline characteristics of patients were summarised by category using percentages for categorical data and either mean and standard deviation (SD) or median and interquartile range (IQR) for continuous variables. Group comparisons were performed using the Kruskal–Wallis test for continuous variables, and either the χ^2^ test or Fisher exact test for categorical variables. The Cochran–Armitage test analysed the trend of anaemia prevalence by age group. Multivariate logistic regression was performed to identify factors associated with anaemia, presenting adjusted odds ratios (AOR) with 95% confidence intervals (CIs). A *P* value <0.05 was considered statistically significant. Age was modelled categorically and as a non-linear continuous variable using spline terms. Potential effect modification by sex was explored through interaction terms. Data were analysed using R (version 4.0; http://www.r-project.org/). Additional sensitivity analyses were conducted to determine whether longer periods of follow-up, 6 months, and 12 months, respectively, following the diagnosis of anaemia, would result in greater testing rates and higher prevalence estimates.

## Results

### Cohort description

Of the 112 181 patients included in this study, the mean age was 54 years and 54.7% were women. Overall, 13 441 patients (12.0%) were classified as anaemic. Compared with patients without anaemia, patients classified as anaemic were on average older (63.4 years versus 52.9 years), had significantly lower levels of kidney function by eGFR or creatinine (*P*≤0.001) and serum albumin concentrations (*P*≤0.001), and had significantly higher levels of inflammatory markers (*P*≤0.001) and glycaemia (*P*≤0.001) (Table 2).

**Table 2.** Baseline characteristics of patients by anaemia status

Variable	*n*	Overall	No anaemia	Anaemia	*P* value
Observations, *n* (%)	112 181	112 181	98 740 (88.0)	13 441 (12.0)	
Age at baseline, years, mean (SD)	112 181	54.1 (17.5)	52.9 (16.9)	63.4 (19.3)	<0.001
**Age group, years**					<0.001
<45		36 351 (32.4)	33 465 (33.9)	2886 (21.5)	
45–64		43 660 (38.9)	40 390 (40.9)	3270 (24.3)	
65–74		18 735 (16.7)	15 942 (16.1)	2793 (20.8)	
>75		13 435 (12.0)	8943 (9.1)	4492 (33.4)	
**Sex**					<0.001
Female		61 402 (54.7)	53 284 (54.0)	8118 (60.4)	
Male		50 779 (45.3)	45 456 (46.0)	5323 (39.6)	
**Clinical setting^a^**					<0.001
Emergency department		11 120 (9.9)	9074 (9.2)	2046 (15.2)	
General practice		87 947 (78.4)	79 652 (80.7)	8295 (61.7)	
Inpatient		5943 (5.3)	4204 (4.3)	1739 (12.9)	
Outpatient		7171 (6.4)	5810 (5.9)	1361 (10.1)	
**Markers of kidney function**					
Urea, mmol/l, median (IQR)	73 045	4.7 (2.2)	4.7 (2.0)	5.3 (3.6)	<0.001
Serum creatinine, μmol/l, median (IQR)	112 181	73.0 (21.0)	73.0 (19.0)	76.0 (35.0)	<0.001
eGFR ^b^,ml/min/1.73 m^2^, median (IQR)	112 181	91.0 (26.5)	91.9 (24.9)	79.8 (43.1)	<0.001
**eGFR category, ml/min/1.73 m^2^**					<0.001
>90		58 296 (52.0)	53 507 (54.2)	4789 (35.6)	
60–90		42 975 (38.3)	38 296 (38.8)	4679 (34.8)	
30–59		9748 (8.7)	6516 (6.6)	3232 (24.0)	
<30		1162 (1.0)	421 (0.4)	741 (5.5)	
**Inflammatory markers**					
White blood count, 10^9^ /l, median (IQR)	111 837	6.4 (2.7)	6.4 (2.6)	6.6 (3.3)	<0.001
Platelet count, 10^9^ /l, median (IQR)	111 843	247.0 (84.0)	246.0 (81.0)	262.0 (114.0)	<0.001
C-reactive protein,^c^ mg/l, median (IQR)	8076	9.0 (16.0)	8.0 (12.0)	18.0 (46.0)	<0.001
**Red blood cell indices**					
Haemoglobin, g/dl, median (IQR)	112 181	13.9 (1.8)	14.1 (1.6)	11.7 (1.0)	<0.001
Red blood count, 10^12^ /l, mean (SD)	112 160	4.5 (0.5)	4.6 (0.4)	3.9 (0.5)	<0.001
Red cell distribution width, %, median (IQR)	89 176	13.5 (1.0)	13.4 (0.9)	14.3 (2.0)	<0.001
MCH, pg, mean (SD)	112 155	30.5 (1.9)	30.7 (1.6)	29.3 (3.1)	<0.001
MCHC, g/dl, mean (SD)	112 180	33.7 (1.1)	33.8 (1.0)	32.9 (1.2)	<0.001
MCV, fl, mean (SD)	112 181	90.6 (5.1)	90.8 (4.6)	89.0 (8.0)	<0.001
**Iron status, B12, and folate levels^c^**					
Serum iron, μmol/l, mean (SD)	6315	20.0 (7.9)	20.8 (7.5)	12.3 (7.5)	<0.001
Ferritin, ng/ml, median (IQR)	28 476	87.0 (128.0)	91.0 (128.0)	43.0 (116.8)	<0.001
Transferrin, g/l, mean (SD)	6342	2.5 (0.4)	2.5 (0.4)	2.6 (0.6)	0.101
TIBC, μmol/l, mean (SD)	6342	63.6 (10.6)	63.4 (9.8)	65.2 (16.3)	0.101
Transferrin saturation, %, median (IQR)	6296	31.1 (16.6)	32.0 (16.1)	19.4 (18.6)	<0.001
B12, ng/ml, median (IQR)	8405	368.0 (176.0)	369.0 (174.0)	363.0 (192.0)	0.224
Folate, ug/l, median (IQR)	7294	8.9 (6.1)	9.0 (6.0)	8.1 (6.3)	<0.001
**Nutritional and metabolic markers**					
Serum albumin, g/l, mean (SD)	76 551	40.0 (3.7)	40.5 (3.3)	36.5 (4.7)	<0.001
Serum calcium, mmol/l, mean (SD)	22 779	2.3 (0.1)	2.3 (0.1)	2.3 (0.1)	<0.001
Serum phosphate, mmol/l, mean (SD)	19 618	1.1 (0.2)	1.1 (0.2)	1.2 (0.2)	<0.001
Serum potassium, mmol/l, median (IQR)	64 338	4.4 (0.6)	4.4 (0.6)	4.3 (0.7)	<0.001
**Lipid markers**					
Total cholesterol, mmol/l, mean (SD)	18 590	5.1 (1.0)	5.1 (1.0)	4.6 (1.0)	<0.001
Triglycerides, mmol/l, median (IQR)	30 086	1.2 (0.8)	1.2 (0.8)	1.1 (0.7)	<0.001
**Glycaemic status**					
History of diabetes, %^d^		12 130 (10.8)	9274 (9.4)	2856 (21.2)	<0.001
Glucose, mmol/l, median (IQR)	33 344	5.0 (0.9)	5.0 (0.9)	5.1 (1.3)	<0.001
HbA1c DCCT, %, median (IQR)	16 382	6.0 (1.3)	5.9 (1.2)	6.5 (1.6)	<0.001
**Markers of liver function**					
Alkaline phosphatase, IU/l, median (IQR)	102 049	68.0 (27.0)	68.0 (26.0)	71.0 (35.0)	<0.001
Alanine transaminase, IU/l, median (IQR)	101 429	22.0 (13.0)	22.0 (14.0)	18.0 (10.0)	<0.001
GGT, IU/l, median (IQR)	101 751	21.0 (19.0)	21.0 (19.0)	19.0 (20.0)	<0.001
Total bilirubin, μmol/l, median (IQR)	12 907	11.0 (7.0)	11.0 (6.0)	9.0 (6.0)	<0.001

^a^ Clinical setting refers to the location of patient when the laboratory test was taken. ^b^ eGFR: estimated glomerular filtration rate (ml/min per 1.73 m^2^) was based on the Chronic Kidney Disease Epidemiology Collaborative (CKD-EPI) equation. ^c^ First available measurement within 90 days of baseline assessment. ^d^ History of diabetes defined as HbA1c>6.5% or fasting plasma glucose >7.0 mmol/l on two consecutive tests before index date. DCCT = Diabetes Control and Complications Trial. GGT = gamma-glutamyl transferase. MCH = mean corpuscular haemoglobin. MCHC = mean corpuscular haemoglobin concentration. MCV = median corpuscular volume. TIBC = total iron-binding capacity.

### Prevalence of anaemia

**Figure 1.**
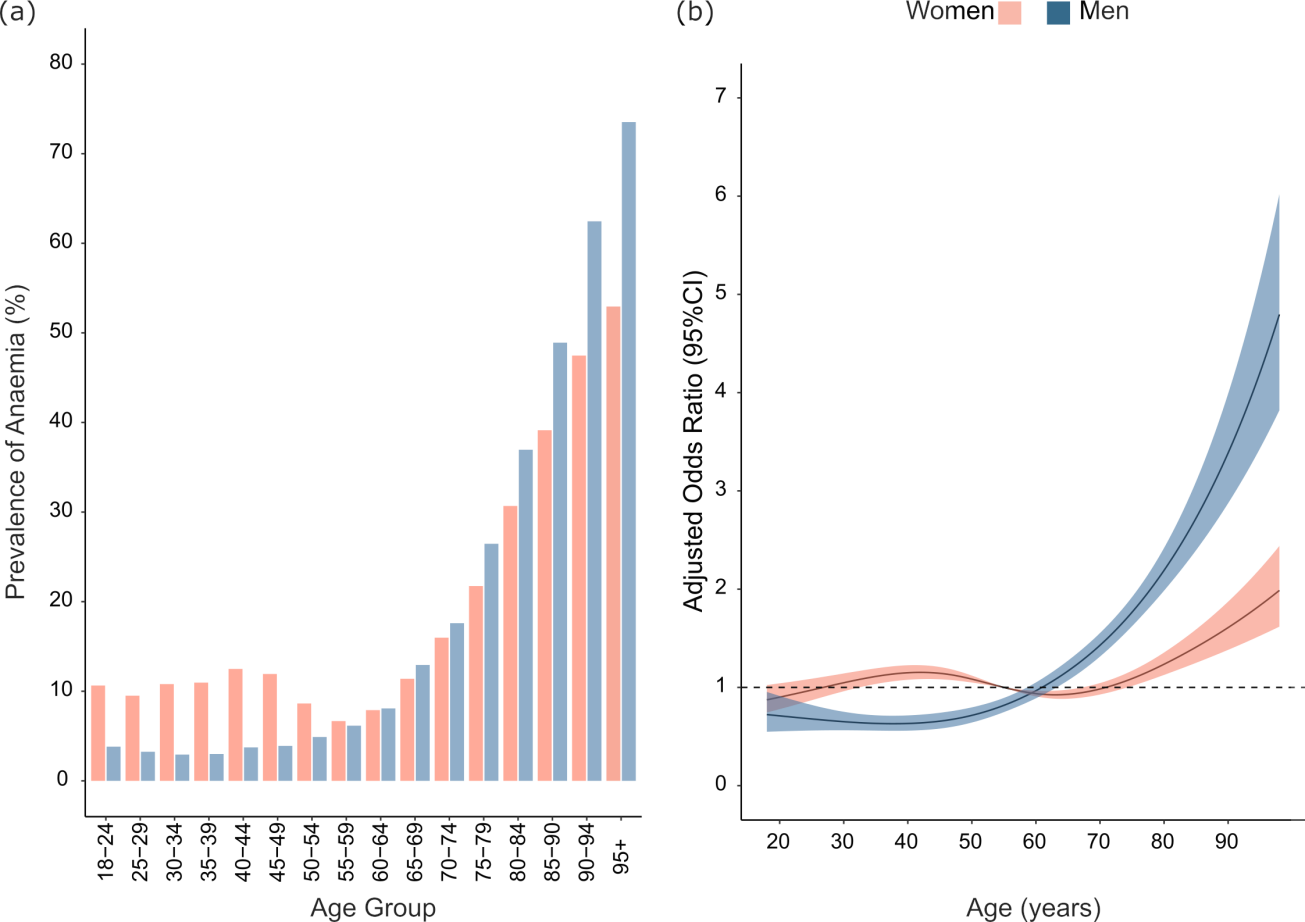
(**A**) Prevalence of anaemia by age group and sex. (**B**) Adjusted odds ratio of anaemia by age, sex with age modelled as a non-linear variable and an interaction term between age and sex was included in the final model. (Reference point: women aged 55 years). Final model was adjusted for age, sex, eGFR category, clinical setting, diabetes, white blood cell count, alanine transaminase, alkaline phosphatase, and serum albumin

The overall prevalence of anaemia was 12.0% (95% CI = 11.8% to 12.2%) (Table 3). The majority of patients had a normocytic anaemia (79.2%), 14.8% had macrocytic, and the remaining 5.9% had a microcytic anaemia. The prevalence of anaemia was 10.5% in men (95% CI = 10.2% to 10.8%), 13.2% in women (95% CI = 13.0% to 13.5%), and 12.9% in women attending maternity services (95% CI = 9.2% to 17.8%) (Table 3). The prevalence of anaemia was significantly higher in women than men up to the 55–59 year age category (*P*<0.001), after which age, anaemia was more common in men (Figure 1a). Prevalence increased more rapidly with age in men than in women. A J-shaped relationship between age and anaemia was found in women. Greater differences in the prevalence of anaemia between the sexes were seen in the younger age categories.

**Table 3.** Prevalence of anaemia by sex, clinical setting, and kidney function

Population	Cases	Total	Overall (95% CI)	Mild anaemia^a^ (95% CI)	Moderate anaemia^b^ (95% CI)	Severe anaemia^c^ (95% CI)
Overall	13 441	112 181	12.0 (11.8 to12.2)	9.1 (8.9 to 9.3)	2.7 (2.6 to 2.8)	0.2 (0.2 to 0.2)
**Sex**						
Male	5323	50 779	10.5 (10.2 to 10.8)	8.7 (8.5 to 8.9)	1.6 (1.5 to 1.7)	0.2 (0.1 to 0.2)
Female	8085	61 147	13.2 (13.0 to 13.5)	9.4 (9.2 to 9.7)	3.6 (3.4 to 3.7)	0.2 (0.2 to 0.3)
Women attending maternity services	33	255	12.9 (9.2 to 17.8)	2.4 (1.0 to 5.3)	10.6 (7.2 to 15.2)	–
χ^2^ *P* value^d^			<0.001	<0.001	<0.001	0.098
**Clinical setting**						
General practice	8295	87 947	9.4 (9.2 to 9.6)	7.6 (7.4 to 7.7)	1.8 (1.7 to 1.8)	0.1 (0.1 to 0.1)
Emergency room	2046	11 120	18.4 (17.7 to 19.1)	13.2 (12.6 to 13.9)	4.7 (4.3 to 5.1)	0.5 (0.4 to 0.7)
Outpatient	1361	7171	19.0 (18.1 to 19.9)	13.5 (12.7 to 14.3)	5.2 (4.7 to 5.7)	0.3 (0.2 to 0.4)
Inpatient	1739	5943	29.3 (28.1 to 30.4)	18.3 (17.3 to 19.3)	10.3 (9.5 to 11.1)	0.7 (0.5 to 0.9)
χ^2^ *P* value^d^			<0.001	<0.001	<0.001	<0.001
**eGFR category**						
>90 ml/min/1.72 m^2^	4789	58 296	8.2 (8.0 to 8.4)	6.5 (6.3 to 6.7)	1.6 (1.5 to 1.7)	0.1 (0.1 to 0.2)
60–90 ml/min/1.72 m^2^	4679	42 975	10.9 (10.6 to 11.2)	8.7 (8.4 to 8.9)	2.1 (1.9 to 2.2)	0.2 (0.1 to 0.2)
30–59 ml/min/1.72 m^2^	3232	9748	33.2 (32.2 to 34.1)	23.2 (22.4 to 24.1)	9.5 (8.9 to 10.1)	0.5 (0.3 to 0.6)
<30 ml/min/1.72 m^2^	741	1162	63.8 (60.9 to 66.5)	34.9 (32.2 to 37.8)	27.0 (24.5 to 29.7)	1.8 (1.2 to 2.8)
χ^2^ *P* value^d^			<0.001	<0.001	<0.001	<0.001

^a^ Mild Anaemia WHO definition, men: Hb 11–12.9 g/dl, women: Hb 11–11.9 g/dl, pregnant women: 10–10.9 g/dl. ^b^ Moderate anaemia WHO definition, men: Hb 8.0–10.9 g/dl, women: Hb 8.0–10.9 g/dl, pregnant women: 7.0–9.9 g/dl. ^c^ Severe anaemia WHO definition, men: Hb <8 g/dl, women: Hb <8 g/dl, pregnant women:<7 g/dl. ^d^ χ^2^ tested for differences in categories of anaemia severity by sex, clinical setting and eGFR group. eGFR = estimated glomerular filtration rate

The prevalence of anaemia was greatest among patients classified as inpatient (29.3%), followed by outpatient (19.0%), emergency room (18.4%), and general practice (9.4%) (Table 3). As expected, the prevalence of anaemia increased with worsening kidney function (from 8.2%, 10.9%, 33.2% to 63.8%, respectively, for each lower eGFR category >90, 60–89, 30–59,<30 ml/min/1.72 m^2^, *P*<0.001). The majority of patients were classified as having a mild anaemia (9.1% overall), although inpatients, those with advanced kidney disease, and women attending maternity services experienced a high prevalence of moderate anaemia (10.3%, 27.0%, and 10.6%, respectively).

### Determinants of anaemia

In multivariate analysis, the likelihood of anaemia was significantly lower in men when compared with women (AOR: 0.82 [95 CI = 0.75 to 0.89], *P*<0.001) (Supplementary Table S1). A significant age and sex interaction (*P*<0.001) was found as shown in Figure 1(b). Compared with women aged 55 years, older men were significantly more likely to have anaemia than older women, while younger women had significantly greater odds of anaemia than younger men. A J-shaped relationship was found between age and anaemia in women with the greatest likelihood of anaemia in those aged <50 years and >65 years. As expected, the likelihood of anaemia was greatest for those with the poorest category of kidney function with AOR: 1.91 (95 CI = 1.72 to 2.11) for eGFR 30–59 ml/min/1.72 m^2^ and AOR: 3.89 (95 CI = 3.15 to 4.79) for eGFR <30 ml/min/1.72 m^2^ compared with the referent group >90 ml/min/1.72 m^2^ (AOR 1.00), respectively (Supplementary Table S1). Patients with diabetes experienced almost two-fold higher odds of anaemia compared with those without diabetes (AOR: 1.78 [95% CI = 1.65 to 1.92]). The likelihood of anaemia also differed significantly across clinical care settings. Compared with patients in general practice, the likelihood of anaemia was higher for inpatients (AOR 1.48 [95% CI = 1.34 to 1.63]), outpatients (AOR: 1.28 [95% CI = 1.17 to 1.40]) and for those in the emergency department (AOR: 1.18 [95% CI = 1.08 to 1.30]) (*P*<0.001). The likelihood of anaemia also increased markedly with lower serum albumin concentrations (AOR: 2.30 [95% CI = 2.22 to 2.37]) for every 1 g/dl lower albumin.

### Extent of investigation for vitamin B12, folate, and iron deficiency within 3 months of anaemia diagnosis

During follow-up, only 42.2% of patients with anaemia had a repeat full blood count within 3 months, and 83.2% of them remained anaemic. Screening for deficiencies of iron, B12, and folate was generally low, with limited testing for serum ferritin (33.7%), TSAT and serum iron (11.2% each), and vitamin B12 and folate levels (19.8% and 17.6%, respectively) with CRP measurements available for 21.0% of patients with anaemia. Screening for deficiency was equally low when categorised by severity of anaemia. Although the screening rates increased with increasing severity of anaemia, a large proportion of patients did not receive a screening test for iron, B12 or folate deficiency (Supplementary Table S2).

The prevalence of absolute iron deficiency (AID) was 37.0% and the prevalence of functional iron deficiency (FID) was 26.4% (Table 4). The prevalence of AID increased with anaemia severity (mild: 33.0%, moderate: 44.0%, severe: 68.9%, Supplementary Table S3) and was the most common cause of microcytic anaemia (Supplementary Table S5). The prevalence of B12 and folate deficiency was 6.3% and 5.8%, respectively, increasing modestly with worsening severity of anaemia. B12 and folate deficiency were more common in patients with macrocytic anaemia than normo- or micro-cytic anaemia. Detailed investigations by anaemia type (MCV classification) and severity are provided in Supplementary Tables S2–S5. In sensitivity analyses, where the follow-up periods were extended to 6 months and 12 months, respectively, screening rates improved; however, the overall prevalence of each deficiency remained low as shown in Supplementary Tables S6 and S7. Within 12 months of anaemia diagnosis, 75.0% of patients had a follow-up haemoglobin with median time to follow-up of 70.5 days (IQR: 14–170). Of these patients, 76.4% remained anaemic at the time of follow-up test.

**Table 4.** Extent of investigation for vitamin B12, folate, iron deficiency, and C-reactive protein within 3 months of anaemia diagnosis

Investigation	*n*	Total	Screened, %	Median daysto test (IQR)	Deficiency definition	Prevalence (95% CI)
**Vitamin parameters**						
Vitamin B12	2659	13 441	19.8	2 (0–19)	B12<200 pg/ml	6.3 (5.4 to 7.3)
Folate	2362	13 441	17.6	2 (0–20)	Folate <3 ng/ml	5.8 (4.9 to 6.8)
**Iron parameters**						
Ferritin	4534	13 441	33.7	0 (0–6)		
Serum iron	1505	13 441	11.2	2 (0–19)		
Transferrin	1537	13 441	11.4	2 (0–19)		
Transferrin saturation	1502	13 441	11.2	2 (0–19)		
Absolute iron deficiency	4534	13 441	33.7	0 (0–6)	Ferritin <30 ng/ml	37.0 (35.6 to 38.4)
Functional iron deficiency	4830	13 441	35.9	0 (0–6)	Ferritin 30–99 ng/mlOR	26.4 (25.2 to 27.7)
					Ferritin 100–299 ng/ml AND TSAT <20%	
**Inflammatory markers**						
C-reactive protein	2829	13 441	21.0	0 (0–7)		
Follow-up haemoglobin	5668	13 441	42.2	20 (3–48)	WHO anaemia criteria proportion still anaemic at follow-up	83.2 (82.2 to 84.2)

Days to test is the number of days from date of anaemia diagnoses, 0 represents the same day as anaemia diagnosis. IQR = interquartile range. TSAT = transferrin saturation. WHO = World Health Organization.

## Discussion

### Summary

In this large population-based study, we report a substantial burden of anaemia within the health system in the Republic of Ireland. Anaemia was prevalent in all clinical settings, especially in acute care and outpatient specialist programmes. High-risk vulnerable groups, including patients with advanced kidney disease, diabetes, and older people, were far more likely to have anaemia and should be considered for targeted screening programmes. However, despite the substantial burden of anaemia, screening rates for treatable anaemia subtypes were remarkably low during the 3-month follow-up period with only modest increases at 12 month follow-up. Only about one-third of patients were screened for iron deficiency, and <20% were screened for B12 or folate deficiency. Additionally, 83.2% of patients with anaemia at baseline remained anaemic at follow-up. This study has underscored the high burden of anaemia in the health system, particularly among high-risk groups, and has highlighted the low rates of screening for underlying treatable causes.

This study of 112 181 patients revealed significant variation in anaemia prevalence. High rates were observed in older patients, both men and women, those with diabetes and advanced kidney disease, and specific clinical settings. Prevalence exceeded 50% in older men and women, highlighting their increased vulnerability. Patients with advanced kidney disease had nearly eightfold higher rates compared with those with normal kidney function. Inpatients, outpatients, and emergency department patients had prevalence rates ranging from 18.4%–29.3%, indicating the importance of these clinical settings for high-risk individuals. Collectively, this new data have suggested that the burden of anaemia is extremely high in specific disease groups and in clinical settings and points to an opportunity for more targeted screening programmes.

### Strengths and limitations

This study has several strengths. The large sample size was constituted from all patients within a regional health system. This allowed us to estimate with precision the burden of anaemia in vulnerable subgroups and across a variety of clinical settings including primary care. The availability of a wide range of commonly measured laboratory biomarkers, including nutritional, inflammatory, and kidney disease markers, allowed us to gain insight to the baseline health status of patients and the extent of screening on follow-up. There were also a number of limitations. The population studied was restricted to healthcare users rather than the general population. The proportion of the adult Midwest population represented by this cohort is described in Supplementary Figure S2. Comorbid conditions and medications were not included so their respective impact on anaemia for this population were outside the scope of this study. Information were lacking on other contributors to anaemia such as diet, body mass index, race, and smoking.^18–20^ The extent of investigation for vitamin B12, folate, and iron deficiency before date of inclusion was also lacking. Nevertheless, our analysis represents a real-world study of patients who were screened for anaemia and underlying causes as part of routine clinical care within the health system in the Republic of Ireland, and whose follow-up has shed light into the extent of surveillance of anaemia and key biomarkers across important patient subgroups.

### Comparison with existing literature

A striking finding in this study was the low frequency of screening for common correctable causes of anaemia. Less than 20% of patients with anaemia were screened for vitamin B12 and folate deficiency, and only one-third were screened for iron deficiency during the 3-month follow-up. Despite some improvement with increasing severity of anaemia, approximately 50% of patients with severe anaemia (Hb <8 g/dl) did not undergo screening (Supplementary Table S2). Our findings lend further support to the notion that anaemia and underlying common causes are inadequately screened for in the wider health system, especially among potentially high-risk groups.^12,18–24^ In a large primary care study of older patients aged >65 years with confirmed anaemia, McCartney *et al* reported screening rates of 55% for either iron, B12, or folate deficiency after 2 years of follow-up.^25^ Similarly, low screening rates for iron deficiency are common among patients with anaemia and CKD and heart failure.^11,12^ Taken together, these studies suggest that there is a gap in the care provided to vulnerable patients with diagnosed anaemia and suggest the need for quality improvement programmes to improve screening practices and follow-up treatment protocols.

The role of haemoglobin, as a predictor of health and wellbeing, is clearly established and specific haemoglobin thresholds are recommended for the general population and those with underlying disease.^16,26,27^ Observational studies have confirmed strong independent associations between haemoglobin concentrations and the risk of future hospitalisation, disability, and death.^2–5^ Furthermore, emerging evidence has confirmed that markers of iron deficiency, and folate deficiency are also independently associate with higher mortality, suggesting that the correction of these may lead to significant health benefits.^28–31^ Recent observations have confirmed that correction of key deficiencies of iron and folate predicts better clinical outcomes.^32,33^ Therefore, improved correction of anaemia and common underlying causes should yield better clinical outcomes. Despite these benefits, this study has confirmed that a large percentage of patients remained anaemic within a 3-month timeframe. This may in part reflect the variability in guidelines for the management of anaemia in specific diseases, or the absence of guidelines on frequency of monitoring of haemoglobin for the general adult population.^26,27,34^ International consensus on existing anaemia guidelines and age-specific thresholds for older people linked to key health outcomes may improve the overall management of anaemia for patients.

The reason for sex differences in anaemia prevalence among older adults are unclear. Sex-specific thresholds in older adults have been questioned.^18^ Iron deficiency anaemia (IDA) related to menstruation^35^ and pregnancy^36^ likely contributes to trends in younger females. Higher prevalence in older males, a finding seen in similar studies,^1,19,37–39^ remains unexplained. Despite demonstrated links between haemoglobin and testosterone,^40^ it is unclear how this impacts older males;^18,41^ however, decreasing androgen levels have been linked to reduced haemoglobin.^42^ Chronic disease may contribute, with some studies indicating a higher incidence of cardiometabolic diseases^43^ and cancers^44^ for men in the Irish population. Nutritional deficiencies have been linked to up to one-third of cases,^6^ and other causes, such as gastrointestinal bleeding,^45^ inflammation and chronic disease,^6^ have been identified.

### Implications for practice

This study has revealed a significant burden of anaemia in the health system in the Republic of Ireland across various clinical settings and high-risk groups, including patients with diabetes, chronic kidney disease, and older individuals. The low screening frequency for treatable causes of anaemia, such as vitamin B12, folate, and iron deficiencies, highlights and important gap in care delivery and the need for quality improvement. Variability in anaemia guidelines for specific diseases and the absence of general guidelines contribute to this variability in care. We advocate for targeted screening programmes and continuous quality improvement initiatives in anaemia management to improve clinical care and patient outcomes in our health system.
